# **‘**Resurgent’, ‘twin’ or ‘silent’ epidemic? A select data overview and observations on increasing psycho-stimulant use and harms in North America

**DOI:** 10.1186/s13011-021-00350-5

**Published:** 2021-02-15

**Authors:** Benedikt Fischer, Caroline O’Keefe-Markman, Angelica (Min-Hye) Lee, Dimitri Daldegan-Bueno

**Affiliations:** 1 Schools of Population Health and Pharmacy, Faculty of Medical and Health Sciences, University of Auckland, 85 Park Rd, Grafton, Auckland, 1023 New Zealand; 2 Centre for Applied Research in Mental Health and Addiction, Faculty of Health Sciences, Simon Fraser University, 515 W. Hastings Street, Vancouver, BC V6B 5K3 Canada; 3 Department of Psychiatry, University of Toronto, 250 College Street, 8th floor, Toronto, M5T 1R8 Canada; 4 Department of Psychiatry, Federal University of São Paulo, R. Dr. Ovídio Pires de Campos, 785, São Paulo, SP 05403-903 Brazil

**Keywords:** Psycho-stimulants, Opioids, Co-use, Epidemic, Public health, Interventions, North America

## Abstract

In the early 2000s, increasing prevalence of psycho-stimulant (e.g., crack/cocaine, methamphetamine) use and related harms, including severe adverse health outcomes, was observed among - mostly marginalized - populations of persons using illicit drugs in North America, underscoring an urgent need for interventions options towards improved prevention and treatment. By about 2010, however, the ‘opioid crisis’, featuring unprecedented use and public health burden, had accelerated into full force in North America, largely muting attention to the psycho-stimulant issue until recently. Recent surveillance data on drug use and related mortality/morbidity from the present decade has documented a marked resurgence of psycho-stimulant use and harms especially in at-risk populations, commonly in direct combination with opioids, across North America, resulting in a ‘twin epidemic’ comprised of opioids and psycho-stimulants We briefly review select epidemiological data indicators for these developments from the United States and Canada; in the latter jurisdiction, related evidence has been less prevalent and systematic but corroborating the same trends. Evidently, the (widely ongoing) focus on the ‘opioid epidemic’ as a ‘mono-type’ drug problem has become an anachronism that requires urgent and appropriate correction. We then briefly consider existing, evidence-based options for – prevention and treatment – interventions targeting psycho-stimulant use and harms, which are substantially more limited and/or less efficacious than those available for problematic opioid use, while presenting major gaps and challenges. The observed resurgence of psycho-stimulants may, indirectly, relate to recent efforts towards curtailing (medical) opioid availability, thereby accelerating demand and supply for both illicit opioids and psycho-stimulants. The presently unfolding ‘twin epidemic’ of opioids and psycho-stimulants, combined with limited intervention resources, presents an acute challenge for public health and may crucially undermine actively extensive efforts to reduce opioid-related health harms in North America.

## Background

In the initial decade of the 2000s, a growing body of evidence documented increasing use of psycho-stimulants (e.g., crack-cocaine, methamphetamine) especially among street-involved persons with drug use in North America (i.e., the United States and Canada) in the [1–3]. Moreover, data consistently showed that exposure to psycho-stimulants predicted higher risk for adverse (acute and chronic) health outcomes in the context of drug use. Select studies of marginalized populations with drug use found crack-cocaine use to be increasingly prevalent, if not predominant across Canada. For example, among persons using injection drug in Vancouver, the prevalence of crack-cocaine use had multifold increased to > 40% by 2005 from previous years [4, 5]. Cocaine (injection or inhalation) use – whether by itself or in combination use with other drugs – was found associated with generally worse physical and mental health status compared to non-users, more socio-economic marginalization, and distinctly elevated risk for human immunodeficiency virus/hepatitis C virus (HIV/HCV) infection as well as (fatal and non-fatal) overdose [6–10]. Methamphetamine was identified as a main risk factor for severe mental health problems (including suicide), drug use risk behaviors (e.g., bingeing or equipment sharing), infectious disease (HIV and HCV) transmission, and overdose among young and other high-risk populations with drug use [11–14]. Psycho-stimulant use and its related harms presented itself as an acute, yet widely neglected epidemic of drug use and adverse public health impacts [5, 15]. Given the mounting evidence, yet faced with rather limited intervention options and resources (especially when compared to other drugs, e.g., opioids), intensifying consideration and discussions occurred by around 2010 as to what appropriate treatment and public health measures were for psycho-stimulant use, and how to more effectively design and deliver interventions to at-risk individuals involved with drug use [16–20].

Yet then, a distinct and extreme phenomenon of substance use and health that came to be dubbed the ‘opioid epidemic’ started to gradually unfold in the first decade of the 2000s. Initially facilitated by unprecedented high and rapidly rising levels of prescription opioid dispensing in North America beginning in that decade, potent opioid drugs (initially: morphine, oxycodone, hydromorphone formulations) were amply diverted and available from medical sources for non-medical use, and became the drug of choice, not only for marginalized individuals with drug use but for large general population segments in North America [21–24]. In the US, the opioid epidemic has been characterized by ‘three waves’, although these are not categorically separate but overlapping phenomena; the first of which involved prescription opioids (− 2010), followed by heroin (2010–2012) and illicit/synthetic opioids (2012 onward) [25, 26]. Among street-involved individuals with drug use, the use of heroin – and possibly that of psycho-stimulants - became substantially reduced and widely replaced by use of prescription opioids as the primary substance during the initial phase of the opioid epidemic [27, 28].

The rapidly expanding use of prescription opioids in both street-involved and general populations went hand-in-hand with corresponding, multifold increases in opioid-related morbidity (e.g., hospitalizations, treatment admissions) as well as mortality (e.g., accidental poisoning deaths) in both the United States (US) and Canada [29–33]. By about 2015, amidst select intervention and policy measures implemented to reduce rampant prescription opioid dispensing and harms in North America, a range of highly potent and toxic, illicit/synthetic opioids (e.g., fentanyl) quickly proliferated on the drug markets and became widely consumed, further accelerating opioid-related morbidity and mortality tolls. By 2018, there were an annual 46,802 opioid-related fatalities (combined from both prescription and illicit opioid products) in the United States, an approximately five-fold increase from 2001 [32, 34]; in Canada, similarly, the opioid-related mortality toll had increased to a count of 4372 and a comparable population rate of deaths, in 2018 [35, 36]. In both countries, the persistently high rates of opioid-related mortality, a disproportionate number  of which occurring among young adults, were adversely affecting life expectancy in the general population [37, 38].

The excessive and persistent burden of opioid-related morbidity and mortality led to the implementation and/or expansion of a series of prevention and treatment measures targeting individuals with at-risk opioid use in Canada, including: widespread naloxone availability and distribution (for opioid overdose reversal); officially sanctioned, and informal ‘supervised consumption sites/facilities; ‘drug-checking’ services, an increasingly diverse range of opioid pharmacotherapy options for the treatment of opioid use disorder, and initial efforts of ‘safer opioid supply’ programming that were offered to an increasingly large proportion of the ‘at-risk’ populations involved in opioid use [39–45]. The twenty-first century’s second decade (2010–2019) became the decade of the ‘opioid crisis’. It is not an unreasonable assessment to suggest that attention, focus and efforts by scientists, program/service and policy officials in related fields in Canada was almost exclusively focused on and devoted to the unprecedented problem of opioid use, harms, and finding possible solutions – even though this only occurred with limited success, as key opioid-related use and harm (e.g., mortality) indicators remained high [35, 43, 46].

### The (re-)emergence of psycho-stimulant use and harms

By 2018, global indicators suggested that psycho-stimulant use has steadily increased, with an estimated 0.4% (18.2 million) of the global adult population using cocaine and 0.7% (34.2 million) using amphetamines; regional estimates, however, for both drugs (1.9 and 2.0%, respectively) are highest for North America [47, 48]. An estimated 0.58% (326,000) global all-cause deaths were attributed to amphetamine dependence, and 0.32% (178,000) to cocaine dependence in 2017, with higher regional rates (approximately 1.5 and 2.5%, respectively) for North America [45]. Psycho-stimulant use has been generally confirmed to be associated with a variety of (physical and mental) excess morbidity outcomes [49–51]; specifically, substantial proportions (up to 25–30%) of incident HIV and HCV transmission, depending on region and use prevalence, have been assessed to be globally attributable to psycho-stimulant use [47, 52].

Specifically in North America, there have been indications of rising prevalence of psycho-stimulant use, and of their contributions to major adverse health harms (e.g., overdose deaths), among persons with substance use in very recent years (e.g., since about 2015) [53–55]. Notably, a large extent of this recent resurgence of psycho-stimulant use has occurred in individuals with active opioid use, i.e. as a form of combination or co-occurring use together with opioids – a phenomenon characterized by some observers as the ‘twin epidemic’ of opioid and psycho-stimulant use or as a possible ‘fourth wave’ of the opioid epidemic. This, in practice, has been facilitated by different factors, including changes in the drug supply, mixed substances with or without the user’s knowledge, or the actual, intended co-use of substances [54, 56]. Related indicator data initially came from US, but are increasingly supported by – albeit more limited and less systematic - Canada-based data.

### US-based indicators

Among respondents in the National Survey on Drug Use and Health (NSDUH) representatively covering the US general adult population, past-month methamphetamine use tripled in prevalence – from 9.0% in 2015 to 30.2% in 2017 – among those reporting heroin use [57]. In a cross-sectional study of urine-drug-test (UDT) results from 1 million unique specimens from health care settings (2013 to 2018), UDT positivity rates for nonprescribed fentanyl increased significantly both among the cocaine-positive results (from 0.9% in 2013 to 17.6% in 2018; + 1850%) and among the methamphetamine-positive results (from 0.9% in 2013 to 7.9% in 2018; + 798%) [58]. Among a large sample of urban individuals with substance use with opioid use disorder (OUD) in the mid-west US, 56% used methamphetamine in the past 6 months; > 4 of 5 had initiated their methamphetamine use after starting the use of illicit opioids [59].

In a national US sample (*n* = 15,741) substance treatment service attendees, illicit opioid use increased from 44.8% in 2011 to 70.1% in 2018; past-month use of at least 1 non-opioid drug occurred in nearly all participants (> 90%), with significant increases in methamphetamine (+ 85%), steady rates for cocaine/crack-cocaine use (33%/35%), yet decreases across nonopioid prescription drug classes (range: − 40% to − 68%) [60]. Based on US-based national Treatment Episode Data Set data, the rate of primary heroin treatment admissions co-reporting methamphetamine use increased from 2.1% in 2008 to 12.4% in 2017 (relative percentage increase: 490%; annual percentage change: 23.4%), with increases among all socio-demographic sub-groups. Among individuals with methamphetamine co-use in 2017, 47% reported injecting, 53% reported inhalation or ingestion routes of use [61]. In the US-based National Emergency Department Sample (2006–2016), emergency department (ED) visit rates involving cocaine with opioids increased (annual percentage change; APC) 14.7%/year; ED visits with cocaine without opioids increased 11.3% until 2012 and then stabilized. For psycho-stimulants, ED visit rates with opioids increased 49.9%/year from 2006 to 2011, and then further increased 14.0%/year 2011–2016; ED visits involving psycho-stimulants without opioids increased 13.9% through the period [62]. In a buprenorphine-based treatment sample for opioid use disorder in Washington state (2015–2018), 30% reported concurrent methamphetamine use (past month); methamphetamine co-use was associated with a > 2-fold hazard for treatment discharge [63].

Among the total of 63,632 drug overdose deaths reported in the US in 2016, 27% (17,258) involved psycho-stimulants, comprising a one-year increase of 42% (cocaine: + 52%; other psycho-stimulants: + 33%) for 2015–2016 alone. The contribution of psycho-stimulants to the drug-related death total (70,237) further increased to 34.5% in 2017 (13,942/19.8% cocaine and 10,333/14.7% other psycho-stimulants). Similarly, opioids were involved in 72.7 and 50.4% of cocaine-involved and psycho-stimulant-involved overdose deaths, respectively, in 2017, indicating extensive overlap between the two drug categories. Following declining and bottom-levels for the period of 2008–2013, psycho-stimulant-related overdose deaths have posted substantially increasing levels post-2015 in the US. Specifically, the rate of cocaine-related overdose deaths more than tripled from 1.4/100,000 in 2012 to 4.9/100,000 in 2019, and the rate of psycho-stimulant-related (e.g., methamphetamine, amphetamine, methylphenidate), increased on average by 29% per year from 2012 to 2019 [53, 64–66]. Additional analyses showed that, while psycho-stimulant-related overdose mortality rates substantially varied between US-states, significant increases in mortality rates occurred in 42 states from 2015/16 to 2017/18 alone; the national average of psycho-stimulant overdose mortality cases co-involving opioids was 50% in 2017/18, confirming a high overlap between drug classes for fatality outcomes. [54].

### Canada-based data

Among participants of two long-term prospective cohorts of persons with injection drug use (*n* = 1030) in Vancouver, the rate of crystal methamphetamine use (past 6 months) increased from 19% in 2007 to 36% in 2017 (*p* < 0.001); daily methamphetamine use proportionally increased more markedly, from 1 to 12%, over the same period. Methamphetamine use was found to be independently associated with major adverse health risks or outcomes, including overdose, sex work, paraphernalia sharing, and violence [67]. A study administering rapid urine drug screen tests for eight major substance classes with three prospective high-risk cohorts of participants with (injection and non-injection; *n* = 669) drug use in 2016 in Vancouver reported urine drug screen (UDS) test positivity for cocaine among 53.7% and for amphetamine/methamphetamine among 41.6% of respondents; individuals with both stimulant-type use predominantly co-tested UDS-positive for fentanyl (72.2 and 75.3%, respectively) [68]. Among respondents in an ongoing survey of ‘harm reduction’ services for individuals with drug use at (urban and non-urban) sites across British Columbia, results indicated that methamphetamine was reported as the most commonly used drug by 69% of respondents in 2018, up from 47% in 2015 [55]. Cross-sectional content analyses of drug seizure samples in British Columbia showed that the annual numbers of samples involving fentanyl (5–1997) and heroin (674–2178) multifold increased 2010–2016; fentanyl content samples co-including crack-cocaine (1–179) and methamphetamine (0–174) substantially increased in this period [69].

Data collected on 5657 site visits collected from persons utilizing an overdose prevention site/drug consumption site in Toronto (October 2017–March 2018) documented that the majority of ‘main drug injected’ (> 80%) involved an opioid drug; 4.3% reported crystal methamphetamine, 3.0% crack-cocaine and 1.3% cocaine (these data only for ‘main drug injected’) [70]. In a survey of clients (*n* = 654) and related service visits (March – June 2018) of a new supervised consumption service comprising multiple routes of administration in Lethbridge, Alberta, increasing monthly client rates – from 60 to 70% - utilized inhalation services. 82.0% of inhalation service visits involved methamphetamine, 11.3% opiates and 3.1% crack-cocaine; in the injection facility, 57.1% involved opiates, 29.9% methamphetamine, 12.4% ‘speedballs’ (fentanyl/methamphetamine combination) [71]. Furthermore, recent key informant data on supervised consumption services in Alberta suggested that the most commonly used drug “changed from opioids and cocaine, to methamphetamine” among service users; in addition, many were “polydrug users and were consuming both opioids and methamphetamine” (p.13) [72]. While detailed data are sparse, psycho-stimulants have been reported to dominate illicit drug markets in Eastern Canada, where they appear to be preferred over opioids by persons involved with drug use. Drug content monitoring surveys listed ‘crack-cocaine’, ‘cocaine’ and ‘speed’ as the three drugs reported most frequently used in Montreal [73].

Recent, federally (Public Health Agency of Canada) compiled surveillance data provides (select) information on psycho-stimulant and opioid-related deaths in some [5] Canadian provinces [74]. While totals of stimulant-related deaths were highest in British Columbia (811) and Ontario (887) in 2018, all provinces for which data on such deaths are reported indicate that the majority proportion involved cocaine (range: 54–91%), followed by methamphetamine (35–51%) and other stimulants. In those provinces, the percentage of opioid toxicity deaths where psycho-stimulants were also involved in the death ranged from 31 to 72% in 2018; these ranges were shown to have slightly increased (36–76%) in 2019 [75]. The corresponding national rate for hospitalizations for opioid-related poisonings co-involving poisoning with a non-opioid substance remained steady at 26% from 2016 to 2019. Based on toxicological analyses of 1789 drug overdose deaths involving one or more illicit drug in British Columbia 2015–2017, the majority of cases (86%) included at least one type of opioid (fentanyl in 76% of cases) relevant to death. Non-prescribed stimulants (i.e., methamphetamine/amphetamine, cocaine) relevant to death were identified for 70% of the total of drug fatalities, implying a substantial contribution and co-involvement with opioids towards death [76]. Similarly, toxicological analyses for the total (3.999) of illicit drug-related poisoning deaths in British Columbia 2016–2019, fentanyl and analogues (82.9%), cocaine (49.8%) and methamphetamine/amphetamine (34.2%) products were identified as the main drug classes – alone or in contribution – responsible for fatalities, with fentanyl and methamphetamine involvement indicating increasing trends 2012–2019.

Some additional indicator data have come from the central ‘Prairie provinces’ (e.g. Alberta, Manitoba, Saskatchewan) where a “dramatic rise in methamphetamine use” has been reported, “overtaking [other drugs] as the drug of choice for many” [77, 78]. In Alberta, the rate of ED-visits related to psycho-stimulant use increased by 168%, from 112/100,000 in 2010 to 301/100,000 in 2017. While cocaine-related demand for addiction treatment services remained consistently high, the proportion of clients with crystal methamphetamine use nearly tripled from 2011/12 to 2015/16 [75, 76]. In 2016, 303 (39%) of confirmed drug toxicity deaths were psycho-stimulant-related, while three-quarters (77.2%) co-involved an opioid substance [55]. This trend was underscored by local enforcement data (e.g., from Calgary and Edmonton) indicating multifold annual increases of methamphetamine seizures from 2014 to 2019 [72]. In addition, street-level prices for methamphetamine were reported to be substantially lower than for fentanyl or other opioid products [77, 78]. In Manitoba, the rate of adult clients presenting for addiction treatment reporting methamphetamine use increased from 9% in 2014/15 to 24% in 2018/19, representing the most common primary substance use alongside cocaine, for adults seeking treatment services. Monthly methamphetamine use-related emergency department visits in Winnipeg (Manitoba) increased 16-fold between 2013 (11 visits) and 2017 (187 visits). Furthermore, the rate of illicit drug deaths with methamphetamine identified as primary cause tripled, and the rate of deaths with methamphetamine as contributing cause doubled, between 2016 and 2017 alone [55].

### Epidemiologic implications

A growing body of literature and surveillance data from North America has documented that, after an interim period of decline, the use of and key related harms associated with psycho-stimulants (e.g., amphetamines/methamphetamine, cocaine/crack-cocaine) has been increasing especially among high-risk groups (e.g., street-involved) involved with drug use over the past decade (e.g., post-2010). A large extent of this psycho-stimulant use is not only co-occurring in combination with – an increasingly diverse range (e.g., prescription, natural, illicit/synthetic) - of opioids but has majorly contributed to risks for primary adverse health outcomes, including, for example overdose mortality as well as morbidity (e.g., infectious disease transmission). Data supporting these seismic trends observed – as commonly the case when it comes to surveillance indicators for substance use – are more comprehensive and rigorous from the US, but similarly supported by (somewhat more local/sporadic) evidence from Canada.

A major implication of these trends is that the approximately decade-long ‘silo-’ or mono-centric focus on ‘opioid use and harms’ as North America’s preeminent drug problem/crisis has become a sort of anachronism; rather a type of ‘twin-epidemic’, comprising the main combination elements of opioid and psycho-stimulant use, has gradually emerged and continues to broadly unfold and expand [56, 60]. While opioid-drugs continue to impose a major health and social toll on individuals using drugs and society, psycho-stimulants represent and contribute a substantial adverse impact in these realms, and deserve similar awareness and attention in public discourse, interventions and urgency. This is not currently the case in a corresponding or adequate way. Some of these deficiencies may reflect the socio-cultural status of psycho-stimulants on the low end of the popular hierarchy for drugs and the related attention and interventions they receive.

While the supply-related dynamics behind the ‘opioid crisis’ in North America, and its proposed different supply ‘waves’, are fairly well-documented [25, 26, 79], related supply side drivers may have influenced the observed increases in psycho-stimulant use. First, substantial increases in both global cocaine manufacturing (+ 56% 2013–2016) and amphetamine distribution (+ 20% in seizures 2015–2016 alone) to record levels have been reported and universally increased availability and access for use, while facilitating lower drug prices [47, 80]. In addition, there are ample production (e.g., laboratories) capacities located in close physical proximity (e.g., Mexico) to or established within North America, rendering options for easy and effective supply and related distribution routes [80, 81]. In addition, the surge in psycho-stimulant availability and consumption in recent years may indirectly relate to developments in opioid control and supply in North America post-2012, where ramped up and expanded policy measures and restrictions (e.g., restrictive opioid prescription guidelines and monitoring, enforcement, etc.) have resulted in substantial reductions (up to 50%)  and related supply gaps in general prescription opioid dispensing/availability, and instead shifted supply to and increasing supply of mainly illicit and highly potent/toxic opioid (e.g., fentanyl) products and sources [26, 82, 83]. Similar or related illicit  supply sources and routes may be facilitating access to and availability of illicit opioids as do for psycho-stimulants and their use in present contexts.

Select comparative studies have characterized health and socio-demographic differences between individuals with related drug use. For example, co-users of opioids and psycho-stimulants, or individuals using psychostimulant only, have been found to feature higher rates of chronic co-morbidity (e.g., mental health or infectious disease, including Hepatitis C Virus) and to experience higher rates of social marginalization (e.g., homelessness or incarceration history) than persons involved with opioid-only use, rendering them overall more vulnerable to health-related risks and adverse outcomes (including overdose death) [84–86]. In addition, there may be psycho-behavioral reasons for increasing co-use of psycho-stimulants with opioids. Specifically, persons with long-term use of opioids and their predominant ‘sedation’ or ‘down’ effects may deliberately seek benefits from the complementary stimulating or ‘up’ effects of psycho-stimulants towards a suggested ‘equilibrium’ of drug effects from such co-use. Such desires for and experiences from combined opioid and stimulant use has already been described in earlier (pre-twenty-first century) research on opioid/stimulant co-use patterns when these mainly consisted of heroin and cocaine products [56, 60, 87, 88].

### Implications for interventions

The fundamental epidemiological patterns and shifts behind the increases in psycho-stimulant use and emerging ‘twin epidemics’ of opioids and psycho-stimulants, naturally, have major implications for interventions. In response to the unprecedented opioid crisis and its excessive toll of health and social harms, a wide range of prevention and treatment services specifically targeting risky opioid use have been implemented in North America over the past decade. In Canada, these have included the far expanded availability and distribution of naloxone (for overdose reversal), implementation of numerous, currently involving some 40 sanctioned and several unsanctioned supervised drug consumption sites/services, wide expansion of opioid pharmacotherapy treatment including multiple opioid medications (including injectable opioid) options, ‘drug checking technology’ services, and a series of local ‘safer opioid supply/distribution’ (e.g., hydromorphone, including biometric machine-based distribution) programs, implemented across the country [41, 42, 89–91]. While these interventions have embraced large proportions of persons with ‘at-risk opioid use’, and have been demonstrated to avoid or avert a substantial extent of (e.g., overdose) harms, their overall effectiveness in reducing the adverse opioid-related health burden (e.g., overdose mortality), especially in light of persistently available and hazardous toxic/synthetic opioid drug supply and with the volatile dynamics of the corona-virus disease (COVID-19) pandemic added in 2020, has been limited in Canada [35, 46, 92]. In other words, the ‘opioid crisis’ continues to persist in rather full force, imposing an extensive, and largely unchanged toll of health (e.g., overdose harms) on individuals involved in use and the public.

Unfortunately for the emerging ‘twin epidemic’ scenario, the existent toolbox of available, feasible and especially effective intervention options for psycho-stimulants has presented itself as considerably more limited than that for opioids [47, 93]. For treatment – this in categorical contrast of opioids where opioid-based pharmacotherapy forms the essential, and ‘gold standard’ core of treatment [94, 95] – no medications have been approved, available or have been found convincingly effective for the treatment of either cocaine or amphetamine dependence/disorder, regardless if focusing on withdrawal, abstinence or relapse as main intervention aims [47, 96–98]. A very recent RCT utilizing injectable naltrexone combined with bupropion for methamphetamine use disorder found a relatively higher response rate for the treatment intervention compared to placebo controls, yet a low overall treatment effect (11.1%) [99]. A few select medications (e.g., n-acetyl-cysteine, methylphenidate, modafinil, dexamphetamine) have shown some ‘promise’ for psycho-stimulant dependence treatment, or ‘maintenance’-type interventions, yet require further rigorous investigation and substantially improved evidence for clearer assessment; while some observers are more optimistic regarding the therapeutic potential or prospects of medications-based treatment for psycho-stimulants, others do not expect major advances on the horizon of psycho-stimulant treatment in the foreseeable future [100–102]. The current standard of care for psycho-stimulant dependence treatment primarily involves on psycho-social interventions; however, their effectiveness is limited and commonly does not produce better outcomes than usual care [47, 103, 104]. As somewhat of an exception, the therapeutic application of contingency management intervention components has shown some therapeutic effects towards reducing psycho-stimulant use levels [96, 105–107]; however, this intervention-type is not broadly feasible nor widely implemented in many treatment settings, and so not widely applicable. Beyond the fundamental efficacy for current or prospective treatment options, a systematic review examining barriers to treatment for individuals with methamphetamine use, many expressed low confidence in and reservations regarding the utility and effectiveness of available stimulant treatment options [108].

Cannabidiol (CBD), a cannabinoid with demonstrated anxiolytic, neuro-protective and anti-spasmatic effects, has recently attracted attraction for potential therapeutic usages for substance – and specifically including psycho-stimulant – use disorders [109–111]. While data on some outcomes are mixed and/or substance-specific, recent systematic review data from preclinical and human research indicate that the therapeutic provision of CBD may selectively reverse psycho-stimulant-related toxicity and seizures, behavioural sensitization, motivation to self-administer cocaine and methamphetamine, context- and stress-induced reinstatement of drug seeking behaviours. CBD may also potentiate the extinction and impaired the reinstatement of select psycho-stimulant-induced conditioned place preference (CPP) [112, 113]. While a recent double-blind treatment study of the effects of CBD on crack-cocaine related craving did not find significant effects, longitudinal data from a Vancouver cohort of persons involved with illicit drug use (2012–2015) found that intentional cannabis use was associated with significant reductions in the frequency of crack-cocaine use [114, 115]. Similar general psycho-behavioral benefit effects (e.g., related to withdrawal, aggression, appetite, sleep) derived from cannabinoid co-use have been self-reported by other local – and mostly marginalized – groups of individuals with psycho-stimulant use in a variety of settings [111, 116–118].

For more prevention/public health-oriented services, several community-based interventions have been developed and locally implemented specifically for persons with psycho-stimulant (inhalation) use, many modelled on intervention concepts originally developed for injection drug and/or opioid use. For example, ‘safer crack use paraphernalia’ (SCUP) distribution initiatives became more broadly implemented in Canada a decade ago, with the aims of providing individuals with use with safer stimulant inhalation materials, towards reducing related health (e.g., paraphernalia sharing, infectious disease transmission, oral or pulmonary injuries) risks and to help connect users with health or treatment services; these kits and their distribution have been suggested for expansion to methamphetamine use [17, 119, 120]. Evaluation data for SCUP kits have shown limited uptake and improvements in select health risk behaviors and short-term outcomes among local populations of individuals with psycho-stimulant use, while evidence for sustained or long-term therapeutic impacts is lacking [16, 121–124]. Analogous to the - now broadly implemented - concept of ‘supervised injection facilities’ towards improving public health (e.g., mortality, morbidity) outcomes for persons with injection drug use, there have been longstanding calls to offer similar (‘supervised inhalation’) service interventions to those individuals with psycho-stimulant inhalation use [18, 125, 126]. While substantial proportions of individuals with psycho-stimulant use, and especially those characterized by high-risk for adverse health outcomes, have expressed general interest in the use of such services if available, their implementation in North America has been laggard, partly for regulatory reasons but also due to the particular behavioural profiles of the target population [127–129]. One (sanctioned) supervised inhalation facility was recently implemented in Alberta, select others have been included in (unsanctioned) ‘overdose prevention’ or other supervised consumption facilities [71, 130, 131]. However, besides select descriptive utilization and subjective/experiential data, no rigorous evaluation data, e.g. on health or social outcomes, associated with access or use are available.

Given the high degree of adulteration and contamination of street-level psycho-stimulant supply, and modelled on the concept of ‘safer opioid supply’ initiatives of which some have been locally initiated, some commentators have advocated for ‘safer stimulant supply’ for persons involved with psycho-stimulant use [45, 132–134]. There, however, remain fundamental questions, including general feasibility in respect to the distinct use (‘binge’) profiles that commonly characterize psycho-stimulant use yet also which existing pharmaceutical replacement substances would be suitable for such an intervention, and whether individuals with related substance use would be willing in practice to utilize such programming if offered [132, 134, 135].

Overall, even considering recent – abstract and practical – advances for psycho-stimulant treatment and prevention efforts, it is evident that available options and their effectiveness remain highly limited [47, 93, 96]. Even the more longstanding and broader range and toolbox of interventions for opioid use has proven rather limited in its utility and impact towards reducing the main (e.g., mortality/morbidity) harms, and adverse toll on public health [35, 46]. Given the above-characterized, emerging ‘twin-epidemic’ of psycho-stimulants alongside the persistent ‘opioid crisis’, much of which unfolding in actually combined use and co-occurring harms (e.g., overdose) of the two substance categories among high-risk individuals with use, implies that effective interventions realistically serving and attenuating this problem in combination are even more rare and out-of-reach [76, 136–138]. This is the essential finding of a recent systematic review of pharmacotherapeutic treatment for stimulant use disorders in patients with co-occurring opioid use disorders, concluding that these interventions are essentially non-existent [139].

## Conclusions

Increasing evidence solidly documents an emerging extent and burden of psycho-stimulant use and related harms in North America, including Canada, in recent years. This phenomenon represents both an extension or ‘resurgence’ of a psycho-stimulant crisis initially observed in the early years of the twenty-first century, as well as a ‘twin epidemic’ co-occurring – and likely related causally at least to some degree - with the longstanding and persistent ‘opioid crisis’ that has imposed extensive health and social harm burden across North America. On the individual use-level, a large extent of the ‘twin epidemic’ is unfolding in form of actual ‘co-use’ of psycho-stimulants and opioids, and a likely further elevated risk for adverse health (e.g., overdose/mortality) harm by use of either substance category alone (which is already extensive). A first important implication of this epidemiological situation is that both popular and scientific/specialist perspectives in North America urgently need to be updated and corrected towards acknowledging and focusing on this ‘twin epidemic’, rather than the outdated framing of a singular ‘opioid crisis’. A second crucial implication is  the need for efforts towards the broad-based development and implementation of efforts across the full range (e.g., prevention to treatment) of interventions that effectively aiming and addressing the epidemiological realities of the present co-use/‘twin-epidemic’ scenario. This is much easier advised, or said, than done. As shown, the current toolbox of therapeutic interventions, or even pragmatic public health-oriented measures options for psycho-stimulants is highly limited in availability and efficacy. Even available interventions for psycho-stimulants (e.g., supervised inhalation/consumption as an illustrative example) are commonly considered or applied as secondary or disadvantaged ‘add-on’ type of measure. Beyond, the epidemiological realities of the ‘twin epidemic’ requires intervention efforts that are both feasible and effective for persons with co-use of both substance types, which inevitably renders an even more formidable challenge. Whether ‘resurging’ or ‘twin’ epidemic (or realistically both) – the present scenario of the crisis psycho-stimulant use and harms is equally real and  active in Canada as it is in the US. It requires to be pulled from the silencing shadows of the ‘opioid crisis’, and  addressed with urgent action and progress for interventions. Otherwise, it is possible that the unprecedented health and social harm burdens experienced from and attributed to opioids in North America since the early 2000s will not only persist, but may overall continue to rise due to the increasing hazards and harms added by psycho-stimulants.

## Data Availability

Not applicable.

## Abbreviations

APC: Annual percentage change
CBD: Cannabidiol
COVID: Corona virus disease
ED: Emergency department
HCV: Hepatitis C virus
HIV: Human immunodeficiency virus
NSDUH: National Survey on Drug Use and Health
OUD: Opioid use disorder
RCT: Randomized controlled trial
UDT: Urine drug test
SCUP: Safer crack use paraphernalia
UDS: Urine drug screen
US: United States

## References

[1] Maxwell JC, Rutkowski BA (2008). The prevalence of methamphetamine and amphetamine abuse in North America: a review of the indicators, 1992–2007. Drug Alcohol Rev.

[2] Fischer B, Rehm J, Patra J, Kalousek K, Haydon E, Tyndall M (2006). Crack across Canada: comparing crack users and crack non-users in a Canadian multi-city cohort of illicit opioid users. Addiction..

[3] Roy E, Arruda N, Vaillancourt E, Boivin JF, Morissette C, Leclerc P (2012). Drug use patterns in the presence of crack in downtown Montréal. Drug Alcohol Rev.

[4] Werb D, Debeck K, Kerr T, Li K, Montaner J, Wood E (2010). Modelling crack cocaine use trends over 10 years in a Canadian setting. Drug Alcohol Rev.

[5] Fischer B, Coghlan M (2007). Crack use in north American cities: the neglected 'epidemic'. Addiction..

[6] DeBeck K, Kerr T, Li K, Fischer B, Buxton J, Montaner J (2009). Smoking of crack cocaine as a risk factor for HIV infection among people who use injection drugs. Can Med Assoc J.

[7] Bungay V, Johnson JL, Varcoe C, Boyd S (2010). Women's health and use of crack cocaine in context: structural and 'everyday' violence. Int J Drug Policy.

[8] Tyndall MW, Currie S, Spittal P, Li K, Wood E, O'Shaughnessy MV (2003). Intensive injection cocaine use as the primary risk factor in the Vancouver HIV-1 epidemic. Aids..

[9] Duff P, Tyndall M, Buxton J, Zhang R, Kerr T, Shannon K (2013). Sex-for-crack exchanges: associations with risky sexual and drug use niches in an urban Canadian city. Harm Reduct J.

[10] Roy É, Richer I, Arruda N, Vandermeerschen J, Bruneau J (2013). Patterns of cocaine and opioid co-use and polyroutes of administration among street-based cocaine users in Montréal, Canada. Int J Drug Policy.

[11] Marshall BD, Werb D (2010). Health outcomes associated with methamphetamine use among young people: a systematic review. Addiction..

[12] Wood E, Stoltz J-A, Montaner JSG, Kerr T (2006). Evaluating methamphetamine use and risks of injection initiation among street youth: the ARYS study. Harm Reduct J.

[13] Marshall BDL, Galea S, Wood E, Kerr T (2011). Injection methamphetamine use is associated with an increased risk of attempted suicide: a prospective cohort study. Drug Alcohol Depend.

[14] Fairbairn N, Kerr T, Buxton JA, Li K, Montaner JS, Wood E (2007). Increasing use and associated harms of crystal methamphetamine injection in a Canadian setting. Drug Alcohol Depend.

[15] Department of Justice (2007). Methamphetamine report for federal-provincial territorial ministers responsible for justice.

[16] Malchy LA, Bungay V, Johnson JL, Buxton J (2011). Do crack smoking practices change with the introduction of safer crack kits?. Can J Public Health.

[17] Ivsins A, Roth E, Nakamura N, Krajden M, Fischer B (2011). Uptake, benefits of and barriers to safer crack use kit (SCUK) distribution programmes in Victoria, Canada - a qualitative exploration. Int J Drug Policy.

[18] DeBeck K, Buxton J, Kerr T, Qi J, Montaner J, Wood E (2011). Public crack cocaine smoking and willingness to use a supervised inhalation facility: Implications for street disorder. Subst Abuse Treat Prev Policy.

[19] Degenhardt L, Mathers B, Guarinieri M, Panda S, Phillips B, Strathdee SA (2010). Meth/amphetamine use and associated HIV: implications for global policy and public health. Int J Drug Policy.

[20] Marshall BD, Wood E, Shoveller JA, Buxton JA, Montaner JS, Kerr T (2011). Individual, social, and environmental factors associated with initiating methamphetamine injection: implications for drug use and HIV prevention strategies. Prev Sci.

[21] Mateu-Gelabert P, Guarino H, Jessell L, Teper A (2015). Injection and sexual HIV/HCV risk behaviors associated with nonmedical use of prescription opioids among young adults in New York City. J Subst Abus Treat.

[22] Fischer B, Argento E (2012). Prescription opioid related misuse, harms, diversion and interventions in Canada: A review. Pain Physician.

[23] Nosyk B, Marshall BD, Fischer B, Montaner JS, Wood E, Kerr T (2012). Increases in the availability of prescribed opioids in a Canadian setting. Drug Alcohol Depend.

[24] Roy E, Arruda N, Bourgois P (2011). The growing popularity of prescription opioid injection in downtown Montréal: new challenges for harm reduction. Subst Use Misuse.

[25] Ciccarone D (2019). The triple wave epidemic: supply and demand drivers of the US opioid overdose crisis. Int J Drug Policy.

[26] Fischer B, Pang M, Jones W (2020). The opioid mortality epidemic in North America: Do we understand the supply side dynamics of this unprecedented crisis?. Subst Abuse Treat Prev Policy.

[27] Fischer B, Rehm J, Patra J, Firestone CM (2006). Changes in illicit opioid use across Canada. Can Med Assoc J.

[28] Davis WR, Johnson BD (2008). Prescription opioid use, misuse, and diversion among street drug users in New York City. Drug Alcohol Depend.

[29] Cerdá M, Ransome Y, Keyes KM, Koenen KC, Tracy M, Tardiff KJ (2013). Prescription opioid mortality trends in New York City, 1990-2006: examining the emergence of an epidemic. Drug Alcohol Depend.

[30] Hsu DJ, McCarthy EP, Stevens JP, Mukamal KJ (2017). Hospitalizations, costs and outcomes associated with heroin and prescription opioid overdoses in the United States 2001–12. Addiction.

[31] Jones W, Kurdyak P, Fischer B (2020). Examining correlations between opioid dispensing and opioid-related hospitalizations in Canada, 2007-2016. BMC Health Serv Res.

[32] Gomes T, Tadrous M, Mamdani MM, Paterson JM, Juurlink DN (2018). The burden of opioid-related mortality in the United States. JAMA Netw Open.

[33] Gomes T, Greaves S, Tadrous M, Mamdani MM, Paterson JM, Juurlink DN (2018). Measuring the burden of opioid-related mortality in Ontario, Canada. J Addict Med.

[34] Wilson N, Kariisa M, Seth P, Smith HI, Davis N (2020). Drug and opioid-involved overdose deaths - United States, 2017–2018. Morb Mortal Wkly Rep.

[35] Vojtila L, Pang M, Goldman B, Kurdyak P, Fischer B (2020). Non-medical opioid use, harms, and interventions in Canada – a 10-year update on an unprecedented substance use-related public health crisis. Drugs Educ Prev Policy.

[36] Government of Canada. Opioid-related harms in Canada: Maps Canada: Government of Canada; 2020. Available from: https://health-infobase.canada.ca/substance-related-harms/opioids/maps?index=15 [cited 2020 November, 30].

[37] Dowell D, Arias E, Kochanek K, Anderson R, Guy GP, Losby JL (2017). Contribution of opioid-involved poisoning to the change in life expectancy in the United States, 2000-2015. Jama..

[38] Orpana HM, Lang JJ, George D, Halverson J (2019). At-a-glance - the impact of poisoning-related mortality on life expectancy at birth in Canada, 2000 to 2016. Public Health Agency Canada.

[39] Wood E (2018). Strategies for reducing opioid-overdose deaths - lessons from Canada. N Engl J Med.

[40] Fairbairn N, Coffin PO, Walley AY (2017). Naloxone for heroin, prescription opioid, and illicitly made fentanyl overdoses: challenges and innovations responding to a dynamic epidemic. Int J Drug Policy.

[41] Bruneau J, Ahamad K, Goyer M-È, Poulin G, Selby P, Fischer B (2018). Management of opioid use disorders: a national clinical practice guideline. Can Med Assoc J.

[42] Irvine MA, Buxton JA, Otterstatter M, Balshaw R, Gustafson R, Tyndall M (2018). Distribution of take-home opioid antagonist kits during a synthetic opioid epidemic in British Columbia, Canada: a modelling study. Lancet Public Health.

[43] Strike C, Watson TM (2019). Losing the uphill battle? Emergent harm reduction interventions and barriers during the opioid overdose crisis in Canada. Int J Drug Policy.

[44] Fischer B, Varatharajan T, Shield K, Rehm J, Jones W (2018). Crude estimates of prescription opioid-related misuse and use disorder populations towards informing intervention system need in Canada. Drug Alcohol Depend.

[45] Fischer B, Lee A, Vojtila L (2020). ‘Safer opioid distribution’ as an essential public health intervention for the opioid mortality crisis – considerations, options and examples towards broad-based implementation. Public Health Pract.

[46] Fischer B, Pang M, Tyndall M (2019). The opioid death crisis in Canada: crucial lessons for public health. Lancet Public Health.

[47] Farrell M, Martin NK, Stockings E, Bórquez A, Cepeda JA, Degenhardt L (2019). Responding to global stimulant use: Challenges and opportunities. Lancet (London, England).

[48] Peacock A, Leung J, Larney S, Colledge S, Hickman M, Rehm J (2018). Global statistics on alcohol, tobacco and illicit drug use: 2017 status report. Addiction..

[49] Butler AJ, Rehm J, Fischer B (2017). Health outcomes associated with crack-cocaine use: systematic review and meta-analyses. Drug Alcohol Depend.

[50] Cumming C, Kinner SA, McKetin R, Li I, Preen D (2020). Methamphetamine use, health and criminal justice system outcomes: a systematic review. Drug Alcohol Rev.

[51] McKetin R, Leung J, Stockings E, Huo Y, Foulds J, Lappin JM (2019). Mental health outcomes associated with the use of amphetamines: a systematic review and meta-analysis. EClinicalMedicine..

[52] Cepeda JA, Vickerman P, Bruneau J, Zang G, Borquez A, Farrell M (2020). Estimating the contribution of stimulant injection to HIV and HCV epidemics among people who inject drugs and implications for harm reduction: a modeling analysis. Drug Alcohol Depend.

[53] Seth P, Scholl L, Rudd RA, Bacon S (2018). Overdose deaths involving opioids, cocaine, and psychostimulants - United States, 2015-2016. Morb Mortal Wkly Rep.

[54] Cano M, Huang Y. Overdose deaths involving psychostimulants with abuse potential, excluding cocaine: state-level differences and the role of opioids. Drug Alcohol Depend. 2020. 10.1016/j.drugalcdep.2020.108384 33158665

[55] CCSUA. Changes in stimulant use and related harms: focus on methamphetamine and cocaine (CCENDU bulletin). Ottawa: Canadian Centre on Substance Use and Addiction; 2019.

[56] Ellis MS, Kasper ZA, Cicero TJ (2018). Twin epidemics: the surging rise of methamphetamine use in chronic opioid users. Drug Alcohol Depend.

[57] Strickland JC, Havens JR, Stoops WW (2019). A nationally representative analysis of “twin epidemics”: rising rates of methamphetamine use among persons who use opioids. Drug Alcohol Depend.

[58] LaRue L, Twillman RK, Dawson E, Whitley P, Frasco MA, Huskey A (2019). Rate of fentanyl positivity among urine drug test results positive for cocaine or methamphetamine. JAMA Netw Open.

[59] Daniulaityte R, Silverstein SM, Crawford TN, Martins SS, Zule W, Zaragoza AJ (2020). Methamphetamine use and its correlates among individuals with opioid use disorder in a Midwestern U.S. city. Subst Use Misuse.

[60] Cicero TJ, Ellis MS, Kasper ZA (2019). Polysubstance use: a broader understanding of substance use during the opioid crisis. Am J Public Health.

[61] Jones CM, Underwood N, Compton WM (2020). Increases in methamphetamine use among heroin treatment admissions in the United States, 2008–17. Addiction..

[62] Hoots B, Vivolo-Kantor A, Seth P (2020). The rise in non-fatal and fatal overdoses involving stimulants with and without opioids in the United States. Addiction..

[63] Tsui JI, Mayfield J, Speaker EC, Yakup S, Ries R, Funai H (2020). Association between methamphetamine use and retention among patients with opioid use disorders treated with buprenorphine. J Subst Abus Treat.

[64] Gladden RM, O'Donnell J, Mattson CL, Seth P (2019). Changes in opioid-involved overdose deaths by opioid yype and presence of benzodiazepines, cocaine, and methamphetamine - 25 states, July-December 2017 to January-June 2018. Morb Mortal Wkly Rep.

[65] Kariisa M, Scholl L, Wilson N, Seth P, Hoots B (2019). Drug overdose deaths involving cocaine and psychostimulants with abuse potential - United States, 2003–2017. Morb Mortal Wkly Rep.

[66] Hedegaard H, Miniño AM, Warner M. Drug overdose deaths in the United States, 1999-2019. NCHS Data Brief. 2020;(394):1–8. 33395384

[67] Bach P, Hayashi K, Milloy M-J, Nosova E, Kerr T, Wood E (2020). Characterising the increasing prevalence of crystal methamphetamine use in Vancouver, Canada, from 2006–2017: a gender-based analysis. Drug Alcohol Rev.

[68] Hayashi K, Milloy MJ, Lysyshyn M, DeBeck K, Nosova E, Wood E (2018). Substance use patterns associated with recent exposure to fentanyl among people who inject drugs in Vancouver, Canada: a cross-sectional urine toxicology screening study. Drug Alcohol Depend.

[69] Baldwin N, Gray R, Goel A, Wood E, Buxton JA, Rieb LM (2018). Fentanyl and heroin contained in seized illicit drugs and overdose-related deaths in British Columbia, Canada: an observational analysis. Drug Alcohol Depend.

[70] Kolla G, Kenny KS, Bannerman M, Boyce N, Chapman L, Dodd Z (2020). Help me fix: the provision of injection assistance at an unsanctioned overdose prevention site in Toronto, Canada. Int J Drug Policy.

[71] Bourque S, Pijl EM, Mason E, Manning J, Motz T (2019). Supervised inhalation is an important part of supervised consumption services. Can J Public Health.

[72] Government of Alberta. Impact: A socio-economic review of supervised consumption sites in Alberta. Alberta: Government of Alberta; 2020.

[73] CCSUA. Adulterants, contaminants and co-occurring substances in drugs on the illegal market in Canada (CCENDU bulletin). Ottawa: Canadian Centre on Substance Use and Addiction; 2020.

[74] Government of Canada. Special Advisory Committee on the Epidemic of Opioid Overdoses: Opioids and stimulant-related Harms in Canada 2020 [cited 2021 January, 7]. Available from: https://health-infobase.canada.ca/substance-related-harms/opioids-stimulants.

[75] Government of Canada (2020). Opioid-related harms in Canada.

[76] Crabtree A, Lostchuck E, Chong M, Shapiro A, Slaunwhite A (2020). Toxicology and prescribed medication histories among people experiencing fatal illicit drug overdose in British Columbia, Canada. Can Med Assoc J.

[77] The Canadian Press. 'It's an epidemic:' Inexpensive crystal meth eclipsing opioids on the Prairies Canada: CBC; 2018. Available from: https://www.cbc.ca/news/canada/manitoba/meth-crisis-prairies-1.4881629 [cited 2020 November, 30].

[78] Drug and Alcohol Testing Association of Canada (DATAC). Crystal meth use on the rise in the Prairies Canada. Barrie: DATAC; 2018. Available from: https://datac.ca/crystal-meth-use-on-the-increase-in-the-prairies/ [cited 2020 November, 30].

[79] Mars SG, Rosenblum D, Ciccarone D (2019). Illicit fentanyls in the opioid street market: desired or imposed?. Addiction..

[80] United Nations Office on Drugs and Crime (UNODC). World drug report 2020. Vienna: United Nations Publication; 2020.

[81] Casey B (2019). Impacts of methamphetamine abuse in Canada: report of the standing committee on health.

[82] Fischer B, Jones W, Tyndall M, Kurdyak P (2020). Correlations between opioid mortality increases related to illicit/synthetic opioids and reductions of medical opioid dispensing - exploratory analyses from Canada. BMC Public Health.

[83] Chen Q, Larochelle MR, Weaver DT, Lietz AP, Mueller PP, Mercaldo S (2019). Prevention of prescription opioid misuse and projected overdose deaths in the United States. JAMA Netw Open.

[84] Barocas JA, Wang J, Marshall BDL, LaRochelle MR, Bettano A, Bernson D (2019). Sociodemographic factors and social determinants associated with toxicology confirmed polysubstance opioid-related deaths. Drug Alcohol Depend.

[85] Chawarski MC, Hawk K, Edelman EJ, O’Connor P, Owens P, Martel S (2020). Use of amphetamine-type stimulants among emergency department patients with untreated opioid use disorder. Ann Emerg Med.

[86] Chen L-Y, Strain EC, Alexandre PK, Alexander GC, Mojtabai R, Martins SS (2014). Correlates of nonmedical use of stimulants and methamphetamine use in a national sample. Addict Behav.

[87] Leri F, Stewart J, Fischer B, Jürgen R, Marsh DC, Brissette S (2005). Patterns of opioid and cocaine co-use: a descriptive study in a Canadian sample of untreated opioid-dependent individuals. Exp Clin Psychopharmacol.

[88] Fischer B, Rehm J, Kim G, Kirst M. Eyes wide shut? – a conceptual and empirical critique of methadone maintenance treatment. Eur Addict Res 2005;11(1):1–14. 10.1159/000081410 15608466

[89] Antoniou T, McCormack D, Campbell T, Sutradhar R, Tadrous M, Lum-Wilson N (2020). Geographic variation in the provision of naloxone by pharmacies in Ontario, Canada: A population-based small area variation analysis. Drug Alcohol Depend.

[90] Ivsins A, Boyd J, Mayer S, Collins A, Sutherland C, Kerr T (2020). Barriers and facilitators to a novel low-barrier hydromorphone distribution program in Vancouver, Canada: A qualitative study. Drug Alcohol Depend.

[91] Government of Canada. Federal ations on opioids to date Canada. Ottawa: Government of Canada; 2020 [Available from: https://www.canada.ca/en/health-canada/services/substance-use/problematic-prescription-drug-use/opioids/federal-actions/overview.html.

[92] Gomes T, Khuu W, Martins D, Tadrous M, Mamdani MM, Paterson JM (2018). Contributions of prescribed and non-prescribed opioids to opioid related deaths: population based cohort study in Ontario, Canada. BMJ.

[93] Fischer B, Blanken P, Da Silveira D, Gallassi A, Goldner EM, Rehm J (2015). Effectiveness of secondary prevention and treatment interventions for crack-cocaine abuse: a comprehensive narrative overview of English-language studies. Int J Drug Policy.

[94] Williams AR, Nunes EV, Bisaga A, Pincus HA, Johnson KA, Campbell AN (2018). Developing an opioid use disorder treatment cascade: a review of quality measures. J Subst Abus Treat.

[95] Connery HS (2015). Medication-assisted treatment of opioid use disorder: review of the evidence and future directions. Harvard Rev Psychiatry.

[96] Ronsley C, Nolan S, Knight R, Hayashi K, Klimas J, Walley A (2020). Treatment of stimulant use disorder: a systematic review of reviews. PLoS One.

[97] Castells X, Cunill R, Pérez-Mañá C, Vidal X, Capellà D (2016). Psychostimulant drugs for cocaine dependence. Cochrane Database Syst Rev..

[98] Pérez-Mañá C, Castells X, Torrens M, Capellà D, Farre M. Efficacy of psychostimulant drugs for amphetamine abuse or dependence. Cochrane Database Syst Rev. 2013;9. 10.1002/14651858.CD009695.pub2 PMC11521360 23996457

[99] Trivedi MH, Walker R, Ling W, dela Cruz A, Sharma G, Carmody T (2021). Bupropion and naltrexone in methamphetamine use disorder. N Engl J Med.

[100] Tardelli VS, Bisaga A, Arcadepani FB, Gerra G, Levin FR, Fidalgo TM (2020). Prescription psychostimulants for the treatment of stimulant use disorder: a systematic review and meta-analysis. Psychopharmacology..

[101] Brandt L, Chao T, Comer SD, Levin FR. Pharmacotherapeutic strategies for treating cocaine use disorder—what do we have to offer? Addiction. 10.1111/add.15242 PMC7930140 32888245

[102] Humphreys K. Will hope triumph over experience in pharmacotherapy research on cocaine use disorder? Addiction. 10.1111/add.15266 33078477

[103] Minozzi S, Saulle R, De Crescenzo F, Amato L (2016). Psychosocial interventions for psychostimulant misuse. Cochrane Database Syst Rev.

[104] Crepault JF, Rehm J, Fischer B (2016). The cannabis policy framework by the Centre for addiction and mental health: a proposal for a public health approach to cannabis policy in Canada. Int J Drug Policy.

[105] Shearer J (2007). Psychosocial approaches to psychostimulant dependence: a systematic review. J Subst Abus Treat.

[106] Knapp WP, Soares B, Farrell M, Silva de Lima M. Psychosocial interventions for cocaine and psychostimulant amphetamines related disorders. Cochrane Database Syst Rev. 2007;3.

[107] Lee NK, Rawson RA (2008). A systematic review of cognitive and behavioural therapies for methamphetamine dependence. Drug Alcohol Rev.

[108] Cumming C, Troeung L, Young JT, Kelty E, Preen DB (2016). Barriers to accessing methamphetamine treatment: a systematic review and meta-analysis. Drug Alcohol Depend.

[109] Chye Y, Christensen E, Solowij N, Yücel M. The endocannabinoid system and cannabidiol's promise for the treatment of substance use disorder. Front Psychiatry. 2019;10(63). 10.3389/fpsyt.2019.00063 PMC6390812 30837904

[110] Prud'homme M, Cata R, Jutras-Aswad D (2015). Cannabidiol as an intervention for addictive behaviors: a systematic review of the evidence. Subst Abus.

[111] Fischer B, Kuganesan S, Gallassi A, Malcher-Lopes R, van den Brink W, Wood E (2015). Addressing the stimulant treatment gap: a call to investigate the therapeutic benefits potential of cannabinoids for crack-cocaine use. Int J Drug Policy.

[112] Rodrigues LA, Caroba MES, Taba FK, Filev R, Gallassi AD (2020). Evaluation of the potential use of cannabidiol in the treatment of cocaine use disorder: a systematic review. Pharmacol Biochem Behav.

[113] Calpe-López C, García-Pardo MP, Aguilar MA (2019). Cannabidiol treatment might promote resilience to cocaine and methamphetamine use disorders: A review of possible mechanisms. Molecules..

[114] Meneses-Gaya C, Crippa JA, Hallak JE, Miguel AQ, Laranjeira R, Bressan RA, et al. Cannabidiol for the treatment of crack-cocaine craving: An exploratory double-blind study. Braz J Psychiatry. 2020. [e-pub ahead of print 30 Oct 2020]. 10.1590/1516-4446-2020-1416 PMC8555645 33146345

[115] Socías ME, Kerr T, Wood E, Dong H, Lake S, Hayashi K (2017). Intentional cannabis use to reduce crack cocaine use in a Canadian setting: a longitudinal analysis. Addict Behav.

[116] Gonçalves JR, Nappo SA (2015). Factors that lead to the use of crack cocaine in combination with marijuana in Brazil: a qualitative study. BMC Public Health.

[117] Andrade T, Santiago L, Amari E, Fischer B (2011). ‘What a pity!’ – exploring the use of ‘pitilho’ as harm reduction among crack users in Salvador, Brazil. Drugs Educ Prev Policy.

[118] Lake S, Nosova E, Buxton J, Walsh Z, Socías ME, Hayashi K (2020). Characterizing motivations for cannabis use in a cohort of people who use illicit drugs: a latent class analysis. PLoS One.

[119] Harris M (2020). An urgent impetus for action: safe inhalation interventions to reduce COVID-19 transmission and fatality risk among people who smoke crack cocaine in the United Kingdom. Int J Drug Policy.

[120] Imtiaz S, Strike C, Elton-Marshall T, Rehm J (2020). Safer smoking kits for methamphetamine consumption. Addiction..

[121] Frankeberger J, Cepeda A, Natera-Rey G, Valdez A (2019). Safer crack kits and smoking practices: effectiveness of a harm reduction intervention among active crack users in Mexico City. Subst Use Misuse.

[122] Prangnell A, Dong H, Daly P, Milloy MJ, Kerr T, Hayashi K (2017). Declining rates of health problems associated with crack smoking during the expansion of crack pipe distribution in Vancouver, Canada. BMC Public Health.

[123] Strike C, Watson TM (2017). Education and equipment for people who smoke crack cocaine in Canada: Progress and limits. Harm Reduct J.

[124] Ti L, Buxton J, Wood E, Zhang R, Montaner J, Kerr T (2011). Difficulty accessing crack pipes and crack pipe sharing among people who use drugs in Vancouver, Canada. Subst Abuse Treat Prev Policy.

[125] Cortina S, Kennedy MC, Dong H, Fairbairn N, Hayashi K, Milloy M-J (2018). Willingness to use an in-hospital supervised inhalation room among people who smoke crack cocaine in Vancouver, Canada. Drug Alcohol Rev.

[126] Kerr T, Mitra S, Kennedy MC, McNeil R (2017). Supervised injection facilities in Canada: past, present, and future. Harm Reduct J.

[127] Voon P, Ti L, Dong H, Milloy MJ, Wood E, Kerr T (2016). Risky and rushed public crack cocaine smoking: the potential for supervised inhalation facilities. BMC Public Health.

[128] Speed KA, Gehring ND, Launier K, O’Brien D, Campbell S, Hyshka E (2020). To what extent do supervised drug consumption services incorporate non-injection routes of administration? A systematic scoping review documenting existing facilities. Harm Reduct J.

[129] Watson TM, Strike C, Kolla G, Penn R, Jairam J, Hopkins S (2013). Design considerations for supervised consumption facilities (SCFs): preferences for facilities where people can inject and smoke drugs. Int J Drug Policy.

[130] McNeil R, Kerr T, Lampkin H, Small W (2015). “We need somewhere to smoke crack”: an ethnographic study of an unsanctioned safer smoking room in Vancouver, Canada. Int J Drug Policy.

[131] Foreman-Mackey A, Bayoumi AM, Miskovic M, Kolla G, Strike C (2019). ‘It's our safe sanctuary’: experiences of using an unsanctioned overdose prevention site in Toronto, Ontario. Int J Drug Policy.

[132] Fleming T, Barker A, Ivsins A, Vakharia S, McNeil R (2020). Stimulant safe supply: a potential opportunity to respond to the overdose epidemic. Harm Reduct J.

[133] Tyndall M (2020). Safer opioid distribution in response to the COVID-19 pandemic. Int J Drug Policy.

[134] Ivsins A, Boyd J, Beletsky L, McNeil R (2020). Tackling the overdose crisis: the role of safe supply. Int J Drug Policy.

[135] Sherman SG, Morales KB, Park JN, McKenzie M, Marshall BDL, Green TC (2019). Acceptability of implementing community-based drug checking services for people who use drugs in three United States cities: Baltimore, Boston and Providence. Int J Drug Policy.

[136] O'Donnell J, Gladden RM, Mattson CL, Hunter CT, Davis NL (2020). Vital signs: characteristics of drug overdose deaths involving opioids and stimulants - 24 states and the District of Columbia, January-June 2019. Morb Mortal Wkly Rep.

[137] Jones CM, Bekheet F, Park JN, Alexander GC. The evolving overdose epidemic: synthetic opioids and rising stimulant-related harms. Epidemiol Rev. 2020. 10.1093/epirev/mxaa011 PMC9200066 33511987

[138] Compton WM, Valentino RJ, DuPont RL. Polysubstance use in the U.S. opioid crisis. Mol Psychiatry. 2021;26:41–50. 10.1038/s41380-020-00949-3 PMC7815508 33188253

[139] Chan B, Freeman M, Ayers C, Korthuis PT, Paynter R, Kondo K (2020). A systematic review and meta-analysis of medications for stimulant use disorders in patients with co-occurring opioid use disorders. Drug Alcohol Depend.

